# Locked down: Ontological security and the experience of COVID‐19 while living in poor‐quality housing

**DOI:** 10.1002/jcop.22883

**Published:** 2022-05-24

**Authors:** Philip Brown, Dillon Newton, Rachel Armitage, Leanne Monchuk, Brian Robson

**Affiliations:** ^1^ School of Human and Health Sciences University of Huddersfield Huddersfield UK; ^2^ Policy & Public Affairs Northern Housing Consortium Sunderland UK

**Keywords:** COVID‐19, housing, ontological security, poor‐quality, United Kingdom

## Abstract

The aim of the paper is to illustrate how the housing system in the United Kingdom (UK) has contributed to creating vulnerabilities during the COVID‐19 pandemic. Drawing on the concept of ontological security we look at how living with housing insecurity whilst enduring poor housing conditions has impacted the lives of those living in households. The paper draws on semi‐structured interviews with 50 residents and 8 housing professionals. The findings outline the grinding impact of the pandemic on the ontological security of residents and the coping strategies adopted by a wider range of households who are now increasingly vulnerable. A number of people went into lockdown in vulnerable situations, experiencing deep inequalities and living in poorly maintained homes. This has weakened the ontological security experienced by many households. These represent significant failings of the housing system and housing policy impacting on the health and wellbeing of a wider cohort of people creating additional vulnerabilities.

## 1 INTRODUCTION

The COVID‐19 pandemic has affected and changed the lives of many. However, it has become apparent that some people have experienced the worst impacts of both the pandemic and the responses of governments to control the spread of the virus (Bambra et al., 2020; Belot et al., 2020). Across the world, it has become clear those already vulnerable have been most exposed and disproportionately affected by COVID‐19, highlighting its presence as a syndemic. Those who experienced the worst impacts include those: in receipt of benefits (Sanchez et al., 2021), living with long‐term conditions (Gillard et al., 2021), in precarious employment (Blundell et al., 2020), living in insecure accommodation (Shelter, 2020) and associated poor material conditions (Brown et al., 2021). In the United Kingdom (UK), housing has played a vital role by becoming a key instrument through which the state has tackled the spread of COVID‐19. Indeed, the experience of the pandemic and particular months of ‘hard’ lockdowns in which unprecedented social restrictions were imposed in line with the UK's government message to ‘Stay at Home’ (BBC, 2020 March) will have been highly variable depending on whether people had access safe, secure and decent housing.

Housing is one of the major determinants of health and wellbeing (Marmot, 2010; Rolfe et al., 2020; World Health Organization, 2018), with many people exposed to poorer health outcomes due to the conditions of their homes. The aim of this paper is to show how the UK's current housing system has both compounded existing inequalities and created new vulnerabilities throughout the COVID‐19 pandemic, and in particular during periods in which lockdowns (encompassing stay at home orders, curfews and social distancing) were implemented to flatten the curve of infection rates and hospitalisations. Based on unique new primary research undertaken during the UK's first initial lockdown, at the height of outbreak wave of the pandemic in Europe, we identify the multiple impacts of living with housing insecurity whilst enduring poor housing conditions. Grounding our analysis within the concept of ontological security, we argue that observance of lockdown measures in deleterious domestic environments has amplified and exposed vulnerabilities. The focus of this paper is on the experience of housing in the UK, specifically the North of England. However, we recognise that the presence and persistence of poor‐quality housing transcends international borders and we believe that the findings contained within this paper will have resonance far beyond the study area.

## 2 BACKGROUND

### 2.1 Poor‐quality housing in the UK and its links to vulnerabilities in mental health

Knowledge about the quality of housing in the UK is well established, with data consistently collected as part of annual housing surveys indicating that conditions of dwellings vary considerably across tenures and localities. As a tenure, the private rented sector (PRS) has the highest concentrations of low‐quality housing followed by owner‐occupation. Whilst there are quality issues in the social housing sector, in comparison, it is generally in better condition, with the vast majority of homes meeting the ‘Decent Home Standard’, a government measure that aims to ensure social housing in the UK meets a minimum baseline of decency for tenants (see Department of Community and Local Government, 2006). In contrast, even with the significant increase in newer dwellings over the last decade, the private sector (both rented and owned) remains characterised by older, colder and poorly maintained properties. This is a particularly acute in the North of England which tends to feature a housing stock dominated by concentrations of pre‐1914, low‐value properties, and where around 1 million owner‐occupied and 354,000 private rented housing are estimated to fall below the Decent Homes Standard (The Smith Institute, 2018). Owing to its location in the country, it also tends to experience lower temperatures and wetter conditions than more southerly parts of the UK. As a consequence, there are long‐standing calls for reform of the housing system (McKee et al., 2020; Powell & Robinson, 2019; Powell, 2015) as a result of the economic, social and health inequalities which are created and perpetuated by the current housing system.

In the UK, a great deal of attention has been focussed on exploring and exposing the links between the material quality and conditions of, housing and its implications for public health (see Marmot, 2010). Indeed, it has been estimated that, even before the COVID‐19 pandemic, annual costs of poor housing for the National Health Service are around £1.4billion (Buck & Gregory, 2018). Before the pandemic, various studies had shown that the development of mental ill‐health strongly correlated with housing quality issues such as overcrowding and a lack of space (Barratt et al., 2012), inadequate access to kitchen, bathroom or toilet facilities (ibid), unreliable heating and/or a requirement for additional sources of heat during winter (Liddell & Guiney, 2015), exposure to damp, cold and mouldy conditions (ibid), high levels of noise due to poor sound insulation (Mullings et al., 2013) and general levels of deterioration of the internal and external fabric of dwellings (Rautio et al., 2018). The impact of poor housing quality on children is an area of particular concern (Cross et al., 2021), as it is associated with poor educational attainment and lifelong inequalities in mental and physical health (Alderton et al., 2019). Longitudinal research also points to poor housing quality as a significant causal factor in the development (in both adults and children) of depression (Galea et al., 2005).

At a wider level, there are links between housing affordability and insecurity to mental health issues, with studies linking, financial stressors of fuel poverty (Liddell & Guiney, 2015), rent arrears (Bond et al., 2018) and being behind on mortgage payments (Vásquez‐Vera et al., 2017) to the development and exacerbation of mental health conditions depression, anxiety and stress (Waldron & Redmond, 2017). Un‐elective ‘fixity’, or the feeling of being ‘trapped’ in an inadequate home or neighbourhood, is known to equally exacerbate mental ill‐health (Pearson et al., 2009). Houses in multiple occupation are particularly significant in this respect due to overcrowding and small rooms. A lack of control over shared spaces, recurring maintenance issues and the bureaucracy of negotiating repairs are understood to lead to increased levels of stress (Holding et al., 2020). At the same time, research also highlights that untreated mental health problems can increase the risk of eviction by contributing to behaviours that jeopardise tenancy agreements (Crane et al., 2005), including the non‐payment of rent and issues around the maintenance of dwellings (Stenberg et al., 2009). Far from being abstracted from wider social and political contexts, it is recognised that these issues transcend the domestic boundary and are inextricably linked to the wider contexts of welfare reforms (Power et al., 2014), austerity (Hickman et al., 2018) and job insecurity (Bentley et al., 2016).

Emerging evidence during the pandemic provides further details about the connections between housing and COVID‐19, particularly around the impact on mental health, physical health and the hybridity of work/domesticity. In terms of mental health, researchers cautioned early in 2020 that lockdown for people with limited housing space, a lack of outdoor space, material issues that make housing unfit for habitation, and cramped and overcrowded living arrangements, will likely experience severe impacts on mental health (Gurney, 2020; Holmes et al., 2020). Indeed, research published in June 2020—a month before the easing of the UK's first national lockdown, estimated that 31% of adults in the UK—15.9 million people—had experienced mental or physical health problems during lockdown because of the condition of their housing and lack of space (National Housing Federation, 2020).

Whilst the impact of housing quality on wellbeing are well known, there remains a paucity of knowledge about how both the materialities of housing and the subjectivities of ‘home’ are experienced by households (McKee et al., 2020). Furthermore, it is not known what the emotional consequences are for households who endure poor housing conditions at times of crisis, such as the COVID‐19 pandemic. We address this gap in this paper by drawing on the ‘lived experience’ (McIntosh & Wright, 2019) of those voices of householders living through this context by drawing on the conceptual framework of ontological security.

### 2.2 Housing and ontological security

As McCarthy (2020) has identified, over the last 30 years there has been an increasing interest in the concepts of place, home and identity and their inter‐relationships. In the social sciences, such interest has tended to look at how these concepts relate to the health and wellbeing (psychological and otherwise) of residents. Padgett (2007) has observed that studies of the relationships between housing, health and psychological well‐being can be classified into dealing with three interrelated dimensions: the material benefits of housing as shelter from the elements; health threats associated with substandard housing and neighbourhoods; and, the psychosocial benefits of housing as ‘home’. It is perhaps unsurprising that a great deal of policy, attention and research has been focused on identifying the first and second dimensions, namely the mental health threats and impacts of housing. These are of particular interest to those researchers and policy actors working in or around the field of public health. However, over recent years there has been an increasing amount of attention placed on the third dimension, the subjective sense of ‘home’ and its relationship to psychological wellbeing. A notable and increasing volume of this study has drawn on the theory of ontological security (Hiscock et al., 2001; Padgett, 2007; Rosenberg et al., 2021; Stonehouse et al., 2020).

Giddens (1991: 92) defines ontological security as ‘the confidence that most human beings have in the continuity of their self‐identity and in the constancy of social and material environments’. When Laing (1965) first coined the term, he was interested in understanding the psychological conditions of dissociative personality disorder to identify healthy patients with a clear sense of identity and autonomy. A person can experience ontological security at different moments in time, depending on the ‘ordinary circumstances of everyday life’ (Laing, 1965; p. 42). Giddens (1991, p. 92) explains further that ‘basic to a feeling of ontological security is a sense of the reliability of persons and things’. In this context housing and home can be conceptualised as both material and symbolic spaces (Easthope, 2014), which provides physical ‘shelter' and, at the same time, be embedded with subjective and multiple meanings (McCarthy, 2020). In their study of older adult homeowners, Dupuis and Thorns (1998, p. 29) provide a useful definition or ‘markers’ for when ontological security is met: ‘…when the home is a site of constancy in the social and material environment; the home is a spatial context in which the day to day routines of human existence are performed; the home is a site where people feel free from the surveillance of the modern world, and the home is a secure base around which to construct identities’. As such, ontological security, in the context of housing, is closely linked with notions of agency and control. As Saunders (1990, p. 361) theorised, housing and ‘home’ can provide a basis of ontological security, because at home ‘people feel in control of the environment, free from surveillance, free to be themselves and at ease…in a world that might at times be experienced as threatening and uncontrollable’.

There is a growing body of work which has explored the notion of ontological security in relation to housing in terms of tenure (i.e., owner‐occupation and renting) (Easthope, 2014; Hiscock et al., 2001; Power, 2017). A key feature of Saunders' position is that those households living in tenure with greater control (i.e., owner‐occupiers) have a greater sense of ontological security than renters. Based on a study of three English Towns, Saunders (1989, p. 181) argued his findings about home for owner‐occupiers reflect key components of ontological security: ‘home owners more readily associate positive image of home with the house they live in; they speak readily of a pride of ownership; they associate home more strongly with values such as personal autonomy; they are more likely to see the home as a place where they can relax and “be themselves”; they are much more strongly attached to the houses they live in; they express choice in selection of where they live; they derive satisfaction from working on their homes’. At the time the argument received mixed response, most noticeably criticism for excluding evidence about the experience of social renters (Forrest, 1991) and for overlooking financial reasons for moving into owner‐occupation (Kingston, 1992). In addition, research has highlighted the consequences of low quality and precarious housing for the ontological security of low‐income owner‐occupiers (Colic‐Peisker et al., 2015).

Whilst the theory of ontological security has come under some scrutiny of late (see Gurney, 2021) for drifting from its original definition, a lack of critical reflection by researchers and the potential overstatement of its impact. In this paper, we apply the concept to the experiences of householders who have lived through a unique crisis to explore the extent to which ontological security can help understand how we maintain good mental and physical health when at home (see e.g., Henwood et al., 2018; Hiscock et al., 2001; Padgett, 2007; Rosenberg et al., 2021). Recent work by Gurney (2020) has questioned the potential harm that ‘home’ can cause in a context of COVID‐19 and the associated social distancing and lockdown measures. Gurney (2020) posited that disruption caused by COVID‐19 would compromise the widely reported psycho‐social benefits of home. In this paper, we aim to illustrate how, within the COVID‐19 pandemic, the housing system has impacted on the ontological security of households and thus contributed to creating and amplifying vulnerabilities. Our particular focus is on the impact of poor‐quality and insecure housing, across tenure.

This paper draws on a broader study which adopted an abductive research design which provides a way for researchers to discover and describe the way the social world is experienced and perceived from the ‘inside’ (in this case those people living in poor‐quality housing) by drawing on those with lived experience, and then moving across to consider the accounts of key informants from stakeholder organisations. This moving across from lay explanations to technical descriptions of social phenomena is ‘…the process used to produce social scientific accounts of social life by drawing on the concepts and meanings used by social actors and the activities in which they engage’ (Blaikie, 1993; 176). As outlined by Bowpitt et al. (2011) such an approach ‘…offers the possibility of taking seriously the various understandings of both service users and providers and moving backwards and forwards between their accounts to develop a more comprehensive understanding of relevant issues’ (p. 13).

## 3 METHODS

This paper is based on research conducted between May and July 2020 (Brown et al., 2020) in two major conurbations in the North of England; Greater Manchester and West Yorkshire. An overview of these study locations can be found in Table 1. A challenge from the outset was locating people who were living in poor‐quality conditions during a pandemic. This was a particular challenge due to operating in accordance with social distancing and stay at home orders and using only digital tools to access households who are coping with poor housing, and therefore ‘hidden’ in nature. To navigate these methodological challenges, we promoted the study through networks and organisations. In addition, we worked closely with two external social research agencies to identify and recruit participants to a sample. Therefore, a purposive, and where possible, snowball sampling approach was used to generate the sample for the study.

**Table 1:** Overview of study locations

City‐region	Population	Local authorities	Ethnicity	Median household income	Spatial type
Greater Manchester	2,848,300^a^	10	White (83.81%), Asian (10.15%), Black (2.76%) Mixed (2.26%)^b^	£27,865^c^	Mixed postindustrial, peri‐urban
West Yorkshire	2,300,000^d^	5	White (81.8%), Asian (11.6%), Mixed (2.1%), Black (2.1%), Other (2.4%)^e^	£28,440^f^	Mixed postindustrial, peri‐urban, rural

^a^ Visit North West (2022) Greater Manchester population http://www.visitnorthwest.com/population/greater-manchester/.

^b^ Ibid.

^c^ Greater Manchester Combined Authority (2020) Greater Manchester housing market monitor http://democracy.greatermanchester-ca.gov.uk/documents/s5216/ITEM%206%20Greater%20Manchester%20Housing%20Market%20Monitorv6%20PS.pdf.

^d^ West Yorkshire Combined Authority (2021) West Yorkshire: State of the region report https://www.westyorks-ca.gov.uk/media/7421/west-yorkshire-state-of-the-region-2021-report.pdf.

^e^ HM Government (2018) regional ethnic diversity https://www.ethnicity-facts-figures.service.gov.uk/uk-population-by-ethnicity/national-and-regional-populations/regional-ethnic-diversity/latest#ethnic-groups-by-area.

^f^ Plumplot (2022) West Yorkshire average salary comparison https://www.plumplot.co.uk/West-Yorkshire-salary-and-unemployment.html#:%7E:text=The%20median%20salary%20is%20ranging,%C2%A331.3k%20in%202021.

Semi‐structured interviews were conducted with a total of 58 people. This comprised of 50 residents and 8 housing professionals who were working with issues on the ground. We also used an online survey to obtain data on the experiences of people who did not respond to interview requests. Lastly, we invited participants to submit photographs and images of their housing conditions. In some interviews, these photographs and images were used to guide and prompt responses to interview questions. Full details of the residents who participated in the research are provided in Table 2.

**Table 2:** Participant demographics.

	Number	Percentage of resident participants (*n* = 50) (%)
*Gender*		
Male	18	36
Female	32	64
*Age groups*		
18–29	12	24
30–39	18	36
40–59	18	36
60–70	2	4
*Employment status*		
Employed (full time and part time)	28	56
Unemployed	11	22
Furlough^a^	9	18
Retired	2	4
*Housing tenure*		
Owner occupation	10	20
Private rented	40	80
*Household type*		
Single	15	30
Family	27	53
Cohabiting	5	10
House share	4	7

^a^ Furlough relates to the UK Government's Coronavirus Job Retention Scheme (colloquially known as the ‘furlough scheme'), which paid up to 80 per cent of the wages of individuals unable to work due to the temporary closure of non‐essential businesses.

In each case, before being invited to take part in a formal interview for the study, we undertook screening checks by asking each potential participant to elaborate on the issues they saw as ‘poor‐quality’ to ensure each was reporting issues that would be recognised by the Decent Homes Standard. In some instances, interviews were not carried out with potential respondents as a result of our assessment of their adherence to these criteria. We feel the stories collected are reflective of the broader experience of households living in the private rented and owner‐occupied sectors. This study received full ethical approval from the Research Ethics and Integrity Committee at the University of Huddersfield.

All interviews were conducted using the telephone and recorded. Data were transcribed verbatim, uploaded into QSR NVivo software and analysed thematically in accordance with the principles of Braun and Clarke (2006). We designed a coding frame to increase the efficiency and effectiveness of our thematic analysis. First, all members of the research team were required to read all transcripts in full. This allowed them to become familiar with each transcript and to reflect across the transcripts to inform themes and patterns. Second, the research team then met to discuss their review of the transcripts and collectively began to codesign a coding framework. To ensure clarity on the purpose of each node, they co‐produced a detailed briefing document to outline the types of data to be coded under each node in an attempt to ensure clarity and consistency within and across the nodes and researchers. Third, the nodes were then divided across the research team, so that upon their more detailed review of the transcripts, each researcher coded to a set number of nodes to further enable the systematic coding and sorting of the data. The coders met regularly throughout this process to share and talk through their coding with the goal of achieving consensus across the research team to finalise the coding collectively (Braun & Clarke, 2021). Finally, these codes were reviewed, informed and re‐analysed by drawing on and applying the concept of ontological security.

## 4 FINDINGS

The analysis that follows reflect households' experiences of their dwellings, sense of home, their lived experience of the pandemic and their lives more broadly. In presenting this analysis we draw on the work of Saunders (1990) and Dupuis and Thorns (1998) in relation to the ‘markers’ of ontological security in the context of housing and home (safety, constancy, control and identities). In doing so we have identified four main thematic areas which we will outline, in turn, these are: feeling [un]safe at home; disruptions to constancy and daily routines; coping with poor housing and the search for control; and, the idea of ‘trapped’ identities within the theme of ‘no escape’. Each of these are detailed below by drawing on excerpts from the interviews with participants. Excerpts are provided with a participant code, their gender, age and their household description.

### 4.1 Staying (un)safe at home

A key feature to Saunder's (1990) definition of ontological security was to emphasise the home as a refuge from the outside world. Whilst the UK government utilised the slogan ‘Stay At Home’ to communicate the importance of staying indoors to stay safe and reduce the rate of transmission (BBC, 2020, March), within the accounts provided, it was clear that ‘home’ was not uniformly experienced with many people actively describing their homes as unsafe. Although we did not receive reports of participants experiencing serious health and safety issues, involving gas or electricity, there were a range of factors which highlighted the state of repair of their home which led them to feeling of insecure. Such issues included major structural factors such as persistent leaks from their roofs from general disrepair (including chimney deterioration), gutters that were causing water to penetrate the dwelling and general disrepair in the immediate outdoor environment (i.e., yards and gardens). Leaks from roofs, guttering and bathrooms were reportedly the root cause of people experiencing severe cases of damp and condensation within the property, which caused further internal damage:

*…you literally get in the bath and then downstairs there's like a waterfall because it just starts leaking. They're the days that were really upsetting, where you've had to take time off work and sit down and wait for a repairman and stuff like that. It just feels like it's just magnified now, since we're home all the time, even the children keep saying, 'Mum, we just want to live comfortably now, we just want to have the house redecorated,' because there are like lines on the wall with watermarks, we've got all that on the walls and stuff. So even they're like, we just want to feel comfortable in our home now, not having to wonder is there going to be another leak again. (PRSMANC13, female, 36, family)*

The most pervasive cause of insecurity cited in the accounts related to gas boilers and heating systems described as outdated and unreliable. The potential for these systems to fail, whilst the household was out of work or on the government's furlough scheme, caused respondents a significant amount of stress. Whilst these concerns were shared across the sample, these issues were particularly felt by owner‐occupiers who would have to fund the repair or replacement themselves:

*I think my concern would be if something like my boiler breaks, that's my bigger concern, because pre‐COVID I was kind of hanging on and, oh, I'll save up and then I'll get my boiler changed. Now, I feel quite stressed because I don't know, my job isn't secure and the issue with my boiler is still going to a point where if it breaks at the same time as I lose my job, I need to have a boiler, I need to have hot water. That's sort of more stressful for me, that those things could happen simultaneously. (OOBRAD1, female, 26, single)*

*I guess it's just my disaster‐thinking anxiety head that worries about what if something happens, like the boiler breaking! (OOBOLT1, female, 39, family)*

Moreover, several respondents reporting difficulties maintaining thermal comfort in their homes due to the cost of heating as the following respondent (in full‐time employment) explains. Such examples of people working but in fuel poverty is not uncommon in the literature (Petrova, 2017):

*I don't put my heating on as much as I should do, I make sure my daughter walks around in slippers, dressing gowns, you come into the home, you take your coat off and you put a dressing gown on, so you walk round in a housecoat basically. I sit there in the winter basically in my housecoat with my slippers on, quite often with a hat on with a blanket over me to make sure that I can keep warm, and I've taught my daughter to do the same. On the coldest, coldest nights, we basically we climb into bed just shortly after we've had hot food and stay there in my double bed cuddled up together underneath all the blankets to keep warm. (PRSMANC14, female, 44, family)*

The issue of households feeling cold was a strong feature of many accounts. Coping with the cold was a persistent cause of anxiety, particularly where children were involved, as well as the financial concerns that came from the associated energy costs of heating inefficient dwellings. The experience meant that many households took a more proactive, and occasionally extreme, approach to energy conservation. Examples of being in bed during the day to keep warm, cutting back on food to afford heating and watching the pre‐payment metre were all a feature in the accounts in this study.

In addition, respondents discussed repair issues such as lack of hot water, toilet malfunctions, issues with food preparation areas and integrated appliances as a feature of their lockdown experiences. These were often combined with longstanding issues that had been present before the pandemic. As OOHUDS1 described:

*I've got a hole in my roof that's gradually getting worse. Water pours in. The central heating hasn't worked for 3 years, so I've had no hot water. I'm unable to use the shower because of a physical disability, and they've lost the disabled facility grant to put a disabled shower in. They won't put that in until the roof's been fixed. My front porch door, there's glass coming off it. It's really dangerous. The inner door, it won't lock… My guttering's all falling to pieces at the back. My neighbour's complained because it sprays water onto their property when they hang their washing out… It's cold because the front porch door won't close, and the inner door's got a big space underneath it, so cold comes straight in, and then to heat up I put fire on. There's mice here. I think it's because they're coming in through the access at the front door. My toilet won't flush. (OOHUDS1, male, 55, single*)

Those without access to outdoor space appeared to have felt particularly vulnerable:

‘*My mental health, yes, definitely. I think if I'd had a garden I could have just gone outside and not felt so confined'. (PRSSCAR1, female, 33, single)*.

Where participants had access to gardens and yards it was common, certainly amongst the PRS households, for that space to be in poor condition and, in some cases, arguably unsafe. PRSBOLT1 provides a broadly reflective description of what the immediate garden or yards looked like for PRS households:

*Yes, so I've got a small yard area at the back, which was in quite a poor shape of repair when I moved in. It was really shabby. It was covered in just crap, like rubble and old broomsticks, and tiles and things in the back space. The walls had a shed that had been pulled down and was half fixed to the wall and half not fixed to the wall, so in terms of an outside I've got two sheds, like an old outhouse and a toilet. So I live in a very old mill, cottage‐style property, with like a very old‐fashioned back yard. I think it was an outdoor toilet and an outdoor coal shed, and they're in quite a poor state of repair. There's paint peeling off, the doors don't close properly. So the outside space is not a nice place to be. It is an outside space in terms of it's mine, but you can't really sit out there and relax because the floors are uneven, there's lumps of concrete sticking out of the ground. There's a manhole cover that doesn't fit properly. It's not a very pleasant place to be. (PRSBOLT1, female, 36, single)*

With residents, those who had resources or options, taking action and moving from their homes to other places to escape these environments and provide more security for themselves,

*Yes, the issues I experienced in the living situation, the house, it wasn't very clean the way it's maintained. The outside garden, the drain were blocked. It was a lot of white substance and, yes, the pipes are blocked. Also, damp was in my room where I live but the landlord wasn't taking any notice. There was a lot of rubbish always left behind at the alleyway. Whenever I reported these issues the landlord didn't take any notice. The living conditions weren't ideal or weren't suitable; they were dangerous to my health. He just wasn't listening, so I just didn't know what to do. As a result, that's why I've had to go back and live in my parents' at the time being. I go there during the day and then go back into my flat to sleep during the night because ‐ not the flat but the room because as a result I do feel very unsafe living there. (PRSHUDS1, male, 30, HMO)*

Throughout the interviews, respondents felt their housing situation had either caused or exacerbated mental health problems during the lockdown period. Respondents described feeling depressed at persistent reminders of repair problems they were unable to fixed as a result of the lockdown rules or financial problems caused by pandemic. Respondents described the impact of living with repair issues without a means of respite. As PRSLDS8 described in relation to a sewer pipe that had collapsed, which led to standing water at the rear of their property:

*…it's depressing. It hasn't affected me physically, but mentally, it's just a niggle, it's constantly there. (PRSLDS8, female, 42, family)*

Respondents described an elevated intensity of insecurity—a sense of apprehension, related to their housing situation and lockdown. Although respondents spoke of an anxiety of experiencing and coping with the day‐to‐day lived experience of a pandemic, the interviews uncovered that a considerable proportion of respondents were experiencing anxiety specific to financial concerns regarding their housing situations. These included the onset of redundancy, sudden drops in income and the need to use savings, or debt, to meet their housing costs, and the concern that, should a major housing repair be required, their precarious financial situation would leave them unable to cover the cost:

*Personally, it's… It's made my existing anxiety a lot worse and added a lot of stress because of financial hardship aspect of it and the uncertainty of work moving forwards. (PRSLDS4, male, 24, flat share)*

Similarly, and perversely the impact of increasing cost associated with their utility bills, as a result of staying in the home for extended periods, was another major source of anxiety for respondents. Respondents detailed the costs of energy usage for space heating and appliances, particularly in households with children:

*Well, it's an added stress that you don't need. Being at home more, I'm conscious that I'm spending money that I don't have. Again, it's that I'll go and check how much I've got in the metre, right it's £9, okay, that's £9 of electric, and I've got the kids on Thursday or Friday, so I'll try and push and push and push and push each last minute on those machines until I have a payday, and then I'll top up £30 on both. Then I try and stretch it out further and further and further, whereas before it was stretched, but I knew that I could go to work, use my laptop and charge my phone or the laptop to use at home. I wasn't using the TV throughout the day. I wasn't using the heating to do clothing as much, or cook as much at home. The kids aren't on the TV or the Xbox when she's round here. (PRSLDS3, male, 41, family)*

*Yes, I just feel like because of the lockdown, the days when we are inside the house, the children like to be warm, so I put the heating on a lot more often. I just feel like the costs are just excruciating really, it's very expensive having to keep topping up the heating costs. (PRSMANC13, female, 36, family)*

### 4.2 Disruptions to constancy and new daily routines

Padgett (2007: 1926) argues that ontological security is a key means by which people gain the health benefits of housing, because health and wellbeing ‘arises from a sense of constancy in one's social and material environment which, in turn, provides a secure platform for identity development and self‐actualisation’. Major events that disrupt a sense of constancy and self‐identity have been theorised as undermining an individual's sense of ontological security (Hawkins & Maurer, 2011; Rosenberg et al., 2021), and throughout our study, it was clear the impact of the pandemic had produced a pronounced and life changing effect on our participants:

*I always see quite a few things in my life as a constant, like work is a constant, family is a constant and everything around it is just up and down, relationships, friendships. Whereas now, I feel like nothing is a constant. I don't feel secure in any aspect of my life anymore because we can just quite clearly see it can just change in an instant (OOBRAD1, female, 26, single)*

Constancy has also been linked closely to the daily routines and rhythms of everyday life (Giddens, 1984, 1991), with the implication that disruptions to normal routines impact a sense of constancy. In housing studies, the ontological security derived from routines has been applied to the daily routine of being able to retreat to the private realm away from an outside world perceived as threatening and uncontrollable (Saunders, 1990) However, there were examples from our interviews of respondents struggling to ‘stay at home’ despite the acknowledging dangers of the outside world and the potential harm straying too far from home during a pandemic:

*Well, It's just been difficult not being able to go anywhere or do anything or being allowed more or less outside the boundary of your own home for your own safety. it's been difficult to arrange your day, in respect that normally you'd get up, and you'd go about, if you needed to go out, or go to an appointment at your doctors or a hospital or go to anything, you'd be able to do it, whereas you've not been able to do all those things. So it's been very worrying, but to adapt to a different day of being here every day, it's trying to think of something different to do every day and something to try and keep yourself busy, because to just go outside and be in the home, it gets very depressing and stressful, every day, not really being able to go out and live your life, as such, I'm feeling that there's much more worry and concern (PRSMANC12, female, 49, family)*

The impact of the lockdown and its implications for the well‐being derived from ordinary routines was voiced across the interview transcripts:

*I can't be someone who just sits there and does nothing. That is not me. I've never done that. I needed some sort of routine and activity (PRSBRAD2, 40, female, couple)*

A significant proportion of daily activities are routine actions involving different places, with the particular ‘time‐space paths' through which an individual moves theorised as establishing familiarity and comfort that contributes to ontological security (Giddens, 1984). Many of our participants made clear their time‐space paths of routinised action before the pandemic involved a variety of locales, often in a familiar and constant order that centred on leaving and returning home. Whilst the routines that take place within the material and social environments of home are understood to contribute to ontological security by creating comfort and familiarity (Dupuis & Thorns, 1998), a number of participants described how they were struggling with confinement to home spaces and without the comfort of normal routines that involved other places and settings:

*I used to have the routine of weekdays, getting up, going to work, coming home, tea, bath/shower, chill‐out, bed. Now, it's like, when you're not in work every day, there's no routine really, it's just you get up when you get up. So it's like sleeping patterns, there's nothing to do at home apart from watch TV and/or read, and that's about it. It's like just pottering about in your house, whereas before, I'd be out and about and I might see friends on an evening or friends, go to a friend's after work, that I work with, and have a cup of tea, things like that. So yes, it's just, life in lockdown has just been very, it is restricted, I've not seen anyone, and don't really do much (PRSLDS11, female, 47, family)*

*The biggest thing we've had to do is almost try to force a little bit of routine and variety into what we do because things became quite stale and quite staid quite quickly. I was getting up in one room and going to another room to work and that was basically it, so there's been a big change in that we didn't realise how much we depended on being able to go out, being able to do this, that and the other, being able to go to bars, restaurants, take trips places. Yes, it's trying not to get too into that same four‐walls mindset, which has been very, very hard, especially when lockdown and stuff was at its peak (PRDSHUDS2, male, 29, family)*

A sense of disruption to old routines and the difficulties of adjusting to new ones was particularly pronounced for participants without adequate housing space. Whilst some participants had been redundant or enroled on the furlough scheme, many had worked throughout the pandemic and were therefore adapting to new routines of home‐working. Issues of homeworking was often linked to which there was available housing space to work alongside others:

*I would just have my working day planned out with a lunchbreak in the middle. I've continued that during COVID‐19, but the issue has been that my partner has been working from home, as well. It's been difficult sometimes in relation to not disturbing him while he's on calls, because we don't have another space. I'm working in the bedroom and he's working in the open‐plan kitchen and lounge. We've had to try to coordinate breaks so I can go and make a drink or have some food. It's not always easy because his calls aren't always scheduled, a couple of times he's been doing calls from the bathroom (PRSMANC4, female, 33, couple)*

For many, a lack of sufficient housing space was perceived compounding the difficulties of adjusting to new routines that were involving all of their time spent at home. This was seemingly particularly acute for respondents living in a House in Multiple Occupation (HMO), in particular for one respondent who had taken the precaution of isolating alone and socially distancing from other people living in the accommodation:

*During lockdown, it's been extremely challenging not being able to go outside; isolating yourself in a room; using a smartphone to watch videos, to watch films. Then during the day, not being able to use the bathroom; sharing with other tenants a kitchen; it has been extremely challenging adapting all of these measures. (PRSHUDS1, male, 30, HMO)*

### 4.3 Coping and the search for control

The extent to which housing can maintain a sense of ontological security has been explored through the home as a site to exert control that is missing in other locales. This understood to be an important means through which people feel in control of their environment, with the literature often discussing this in relation to modification and maintenance of homes (Dupuis & Thorns, 1998; Saunders, 1989). However, in the period before COVID‐19, many people had developed ways of coping largely by spending time outside of their home, as a means of asserting control. As PRSMANC14 explains in detail, the finite amount of time they had previously had to spend in their homemade their situation more bearable. However, the imposed lockdown had removed the possibility of exercising their previous coping mechanisms which had added to their sense of insecurity,

*So the more I look at—because, again, I've been spending more time in the home is making me force that reality even more, because obviously for my own mental health and everything—I didn't spend a lot of time in my home, my home was just somewhere where I watched a bit of TV in the evening and I went to bed, and then I got up and I went out. It wasn't somewhere where I spent a lot of time, I spent a lot of time with my friends at their homes, that's where I socialised, I socialised outside of my home. So it was, I knew that some of these problems were there, but it's like anything else, you can gloss over problems in your head, you justify things in your own head, well it's okay because it's only where I sleep, but because I'm literally spending hours and hours and hours in my own home, you notice that every time you walk down the hallway to the kitchen all the wallpaper bubbles off the wall where it's damp behind. (PRSMANC14, female, 44, family)*.

At the time of the interviews, respondents were coping with their homes, often in financially precarious circumstances located within uncertain global, national and local contexts. Against the canvas of not having a secure home environment, this appeared to be having a particular impact,

*…as I said, the pandemic just made my life a lot harder, and because it made my life a lot harder, my own personal anxieties and depression and everything else has just kicked in greater. (PRSMANC14, female, 44, family)*

However, households tended not to be fatalistic and there were numerous examples of attempts by respondents to demonstrate their agency and cope with their day‐to‐day experience. For some people, there were short‐term issues relating to balancing coping with the conditions in their home, working and, in some cases, supporting their children in their schooling from home. Here respondents talked about their attempts at manipulating the spaces of their home to try and demarcate areas for different activities. For many, this meant coping with disruption and added expenses as a result of having to purchase new office equipment for instance. Respondents also described their attempts to reorganise rooms to reduce the impact of external noise, particularly where young children were unable to sleep:

*Well, down to the music, the loud music and things, me and my partner, we take it in turns. So with the 2‐year‐old, we've actually moved the 2‐year‐old downstairs into the living room and one of us will sleep on the sofa just because obviously he's playing music in his bedroom, and his bedroom is next to our bedroom, so when I go downstairs it is a lot quieter. You can still hear it, but it's not as loud, you know, you can't feel the room shaking. So it's like we've now taken it upon us to, we've had to move one of our children out of the bedroom, and then obviously on alternative nights it's either me on the couch or my partner on the couch, just so our 2‐year‐old can go to sleep! (PRSMANC17, female, 21, family)*

The strategies respondents adopted could be seen to be separated into four distinct areas. Firstly, practical strategies—such as capturing leaking water in bowls/pans, deep cleaning to alleviate mould, using earplugs to prevent external noise and ensuring curtains were closed to preserve indoor heat:

*Adapted using old style of toilet and no showers for a few days, so we would just have a wipe, and having to use buckets to fill with water and things, and the heater as well. Luckily, we had fan heaters and electric heating, so we used them and more blankets and wearing warmer clothes, basically. (PRSLDS10, female, 33, family)*

*Not in terms of repairing, just in terms of managing them, so collecting the water when it's coming down the walls, wiping the walls dry and clean. Getting up in the middle of the night to make sure the situation is being handled during the night, so that when we come down in the morning the carpet is not soaking, the water hasn't splashed onto the sockets, which is next to where it drops down. (PRSLDS5, female, 39, family)*

Secondly, spatial strategies—including reorganising rooms within the house (to adapt to working from home or to avoid external noise) as seen in the excerpt above. Thirdly, psychological strategies which centred on attempts to maintain personal wellbeing through accessing greenspace, where possible, and relaxation techniques. Finally, ownership strategies—whereby tenants circumvented their landlords by paying for or conducting repairs on their own.

*In the end, I had to pay for someone to come out and get rid of the mice myself because I can't have mice running about the flipping house… when it was leaking on the roof I had to pay to have tiles put in. I had to pay for them myself for the tiles to be put up because I couldn't wait. I couldn't get hold of her, and it was flooding in and it was really bad, and I thought, I can't leave it like that, so I had to pay someone to come in. (PRSMANC5, male, 62, couple)*

However, despite these pro‐active agentic accounts, the interviews with respondents also uncovered the ways in which residents felt unable to cope with their housing situation during the lockdown period. Respondents conveyed despondency at the lack of control over their living situation, and this was expressed as signs of ontological (in)security:

*There is no coping strategies for being at home during lockdown. As I said, there is no outdoor space, there is no indoor space. Like all these people who've started to do exercise and everything else, and it's like, oh that's great, yes. I'm thinking that if I jump a little bit too hard I might go through the floorboard, so, you know, can't do any of that. (PRSMANC14, female, 44, family)*

*The way it's impacted me is the ability for me to feel comfortable in my own living space in terms of my living situation, being able to feel safe, to feel in control. It's been very challenging and overwhelming for myself. (PRSHUDS1, male, 30, HMO)*

### 4.4 No escape—The experience of trapped identities

The confidence in one's self‐identity is a clear element to Giddens (1991) definition of ontological security. In their study of owner‐occupiers in New Zealand, Dupuis and Thorns (1998) point out that context is important for understanding identity formation in relation to housing, particularly the wider social and material contexts. Perhaps the bleakest theme amongst the findings was concerned with the respondents' reflections on their circumstances in relation to their current housing circumstances and their future life chances. Many respondents described how their employment status had changed because of the pandemic, with many being made redundant, placed on the UK Government's Coronavirus Job Retention Scheme, or having their working hours substantially reduced. As one participant we interviewed explained:

‘*Everything's just gone a bit pear‐shaped to be honest. I lost my job through COVID‐19, along with hundreds of other people and thousands of other people…*’ (PRSLDS6, female, 51, family).

Respondents spoke of their concern that this significant reduction in income and uncertainty about securing future employment could impact upon their ability to pay their mortgage or rental payments. As PRSMANC17 outlines below, in the absence of being able to access resources they were borrowing money from family members,

*…struggling, because our rent's £600, that leaves us with like £304, and you've got to get food for the month but then obviously my partner has a car, obviously a finance car, he's got insurance payments to keep up with. When you've only got £300 to buy food, and pay for someone's car, and then to pay for your electric, and gas, and… Yes, no we're struggling, we're borrowing money here, there and everywhere off family members that obviously are in a position to lend us the money, but even it's getting to a point now where they're turning round to us and saying, 'We can't keep doing this', which we know they can't keep doing it. (PRSMANC17, female, 21, couple)*

Respondents in the PRS explained how they were particularly worried about meeting their housing costs, especially if they lived alone and were reliant on their single income. Many stated that they were prioritising housing costs over other financial outgoings including food. In addition, many explained how they were afraid of asking for flexibility in their rental payments out of concerns it might antagonise landlords and result in the termination of their tenancies. Indeed, being ‘scared' or apprehensive to request a temporary pause or delay in rental payments was a common feature of the interviews:

*Rent takes a big chunk of what we earn, that's what stresses me the most, and as soon as whatever we earn, I want to pay rent first, as much as it stresses me, it takes a big chunk, but it's one thing that I want to pay first because I'm so scared of being evicted in any situation. I'm not being threatened with that by the landlord or whatever because I've never missed my rent payment, I've not fallen into rent arrears because it's something I'm really scared to get into, so I would rather find other ways to deal with the finances, like having two meals a day or just talking to the utility people to say if we could rearrange payment and stuff, but not miss the rent because, as much as we stress financially, we don't want to be stressed being homeless as well. (PRSMANC3, female, 33, family)*

Whilst there were examples of supportive approaches by some landlords, the vast majority of respondents described how landlords and letting agencies had rejected their requests for repairs. Some respondents felt their landlords were using the presence of lockdown measures to postpone or delay repairs; a fear echoed by one professional who worked around resolving landlord‐tenant disputes. For residents in the PRS, this was perceived as continued pattern of inaction on the part of their landlord or as a pattern of substandard repair work. A number of respondents were frustrated that concerns they had reported before the onset of the pandemic had not been dealt with and that they were now living in homes that were in a state of disrepair. Other renters felt ignored,

*Yes, we mentioned the boiler to him and obviously he said he would get someone to come and have a look at it but then because it was ‐ I think it was the end of April that it happened. So obviously it was like no one could come and sort it out or anything, and then it's kind of been brushed under the carpet, I think. We've addressed it to him, so he knows the problem's there, he knows it needs to be sorted, he's told us it's going to get sorted, but it's like he hasn't said when. (PRSMANC17, female, 21, family)*

*When I rented this, the landlord, about 6 months later, he died. Well, his wife doesn't want anything to do with it. You know what I mean? She's quite happy to get my rent, but she won't do anything that's wrong with it. I back on to a railway and all the fencing is falling down. It's just one thing after another wrong with it and they just don't seem to want to do it. (PRSMANC5, male, 62, couple)*

For some renters, additional impediments to solving housing issues were constraints imposed by tenancies and concern that repair or maintenance work conducted without permission would be in breach of tenancy contracts. Some renters described how they had proposed to carry out work on behalf of landlords, but issues they were experiencing were deemed by landlords themselves as not substantial enough to permit repairs. Other respondents were cautious about fixing issues themselves or through contractors because they felt the costs and accountability lay with landlords.

Most palpable from the data was the distinct sense of fear and dread emerging from the accounts given by households, which pervaded conversations around repairs that properties required during lockdown. Several respondents repeatedly stated that they were hesitant to raise issues and request landlord repairs for concern that it would trigger rent increases, revenge evictions or their landlord to sell their home:

*It does feel like if you ask for anything doing, then they tend to put the price up, which happened last year, unfortunately. (PRSMANC6, female, 43, family)*

*…even though it's damp, I have got somewhere, and I think, it's just, I don't know, I just, I'd be scared if I had to find somewhere else because I wouldn't want to have to pay any more. I think £600 is top lot really, even though there's problems, and I'm scared of complaining to her. (PRSMANC5, male, 62, couple)*

*It's always a matter of is he just going to say, 'Sod it; I'm just going to sell up'? There's always that worry. It's not our house. There's no control over that. (PRSBRAD2, female, 40, couple)*

In some cases, the intense period of lockdown spent residing with poor‐quality housing conditions had a deeper impact. One respondent (PRSBOLT1) felt her confinement in non‐decent conditions was representative of her recent perceived employment failures after losing her job. After spending a lengthy period of confinement in cold conditions within her home (PRSMANC14) described how it had shaped her self‐perception that low‐quality housing was the type of accommodation she could only ever afford would be perpetually limited to:

*Yes, of course. I think because of the poor quality in the house and knowing that my family and friends and people I know, were in nicer accommodation and properties. Knowing I was stuck in somewhere with mould and damp and not much outside space and things like that, it did make me feel down because there was no choice to move or anything. I felt really isolated and stuff, a bit trapped (PRSLDS9, female, 40, lone parent)*

*Yes, every time I walk through the front door I can smell ‐ damp is the first thing I can smell, like washing that hasn't come out of the machine properly. It makes me a little bit paranoid that when people are coming over they think that I don't live very cleanly…I think in terms of my health it really has just*—*it's driven me mad. It's driven me really, really mad. I'm really frustrated and really upset at times, actually. All I want is a nice house to live in. I pay a bloody fortune to live here, and all I want is for it to be nice, and it doesn't feel like a nice house. (PRSBOLT1, female, 36, single)*

## 5 DISCUSSION

This paper has illustrated the relationship between our homes, health, security and sense of wellbeing. These factors are inextricably linked. Adopting the lens of ontological security has enabled us to explore the experiences of households and see where there is emerging precariousness and vulnerabilities. We have attempted to show that although poor‐quality housing is often highly visible, due to observable deterioration evident from the exterior, in many cases non‐decent housing hides in plain sight and is a feature across different tenures. Whilst this is, in itself, not new these issues took on a new meaning and had new impacts during the COVID‐19 pandemic. Incapable of leaving their accommodation in accordance with the UK Government's ‘Stay at Home' rules, poor housing conditions had been a dominant and significant feature of the lockdown experiences of many respondents. This was owed to the abrupt and unanticipated amount of time they had spent in substandard housing environments, removed from their normal routines. We have shown that Dupuis and Thorns (1998) markers of ontological security are frequently not met. Alongside these markers, our findings illustrate that homes have not provided the material constancy that should be expected, where day‐to‐day routines are not able to be performed, where people do not feel in control of their homes, and where their home feels very insecure. Whilst the effects of the global pandemic cannot be understated the findings suggest that the ontological security of residents, across tenure, is fragile. Although this is particularly acute for those in the PRS those who are owner‐occupiers are not insulated from insecurity. Our findings support the assertions by Hiscock et al. (2001) and responding to criticisms posed by Gurney (2021), that the experience of ontological security is more nuanced than being dependent on tenure. It was clear that regardless of tenure, issues such as poverty and wealth, the quality of the area in which respondents live, the quality of the relationships people are a part of and the security of employment are key factors which influence our ontological security. However, it was clear that the condition of our homes is major factor which mediate our ontological security due to the impact our homes have in other facets of our lives.

Many residents were living with prevailing housing problems before the pandemic. These varied in seriousness and impact from having to cope with relatively minor cosmetic issues that became more noticeable during lockdown, to major issues stemming from considerable structural faults. Respondents had often been coping with issues for significant amount of time, and for the most part, they described how they felt able to cope. However, not only had people gone into the COVID‐19 pandemic and subsequent lockdown in poor ‘home’ environments, but their home environment had also deteriorated further during lockdown itself. This was most often experienced around issues of thermal comfort, with households routinely reporting a more refined understanding of their energy use driven by their energy vulnerability and exacerbated by the state of repair of their home. There was a recognition by households that they were increasingly vulnerable and subject to a complex interplay of increasing energy costs due to their home environments, the precarity of their employment and the length of occupancy in their home due to the pandemic. It was also widespread for households to voice awareness and anxiety of the anticipated costs of increased energy usage as the year progressed, in particular when energy consumption was anticipated to peak during winter months, tied with trepidation about changing employment practices which may require them to work from homes during this time.

Regardless of tenure, respondents spoke about living in a persistent state of dread and uncertainty. Anxieties were raised about their ability to meet mortgage and rent payments as well as paying bills and other financial outgoings such as food costs. All respondents described how their social lives had been affected by lockdown, and how because they were unable to leave their home to attend work, meet with family and friends and conduct leisure activities, their home had become the sole place where all their activities took place. The experience of containment over an extended period of time in poor‐quality housing was grinding. As such a key feature of the interview data was the self‐reporting of poor mental health.

Where renting households had contacted their landlord, or agency, for support they reported being let down. Indeed, for the vast majority of renters, a sense that reaching out to report repair issues or request improvements would do little or, perversely, increase their precariousness had the effect of preventing the reporting of repairs or improvements required by the household. As a result, many were suppressing the reality of their situation and not reporting issues as much as they would like out of fear of possible reprisals by landlords.

Unlike residents in the PRS who felt they had some option to landlords, owner‐occupiers felt particularly set apart when it came to accessing support that might resolve their housing quality issues. Many owner‐occupiers had the perception that they could not access advice and support because of their tenure, an issue made worse for some owner‐occupiers who were unable to resolve often complicated and worsening housing issues because of their low incomes.

The findings from this study has demonstrated that the COVID‐19 lockdown has shown in the harshest of terms that the UK's housing system is contributing to creating vulnerabilities particularly in terms of mental health and the ability of people to attain ontological security. This study contributes to the literature which has focused on the presence of syndemics (e.g., Rálaigh, 2021). Whilst the study did not look to identify whether poor‐quality housing impacted on the transmission of COVID‐19 the study did illustrate that this wider social context, one punctuated by housing inequalities, is instrumental in exacerbating the impacts arising from COVID‐19.

Many of the wider socioeconomic impacts of the pandemic may take some time to work through the system as people were struggling to pay their bills before the pandemic, homes haven't been significantly improved so they continue to be expensive to maintain, people are more insecure at work, they are more likely to be more unwell and live in greater fear of eviction or rent increase and so it goes on. The findings point to the need for radical reform of our housing system.

There are several policy implications emerging from this study which can be found in Brown et al. (2020). Of these, a number are worthy of highlighting. We need to ensure households have access to financial resources by not inappropriately and insensitively pursuing people for repayment of debts—people were often utilising savings and credit to meet the rising costs of living—as such reclaiming these debts is likely to add to anxiety and increase the number of people who are evicted from their homes. There is a need to reform the privately rented housing market to improve the standard of housing, strengthen the ability of tenants to make complaints and increase their security of tenure. There is also an imperative for health practitioners globally to be more actively engaged in national debates on housing and housing equity to ensure place of residence is seen as part of an integrated preventative support system. Finally, this paper points to all those who are seeking to reduce mental ill‐health—such as community psychologists, health practitioners, social workers and those working in civil society—should recognise the psychological distress caused by these wider social determinants of health and become engaged in tackling dysfunctional housing markets, labour market insecurity, housing insecurity and the destabilising impact of the welfare system.

### PEER REVIEW

The peer review history for this article is available at https://publons.com/publon/10.1002/jcop.22883.

## Data Availability

The data that support the findings of this study are available on request from the corresponding author. The data are not publicly available due to privacy or ethical restrictions.
